# Exploring Telehealth Readiness in a Resource Limited Setting: Digital and Health Literacy among Older People in Rural India (DAHLIA)

**DOI:** 10.3390/geriatrics7020028

**Published:** 2022-03-01

**Authors:** Tshepo Mokuedi Rasekaba, Pratibha Pereira, Vinaya Rani. G, Riya Johnson, Rebecca McKechnie, Irene Blackberry

**Affiliations:** 1 John Richards Centre for Rural Ageing Research, La Trobe University, Wodonga, VIC 3689, Australia; bec.mckechnie@icloud.com (R.M.); i.blackberry@latrobe.edu.au (I.B.); 2 Department of Geriatric Medicine, JSS Medical College, Mahathma Gandhi Road, Mysore, Mysuru 570004, India; ppatta08@gmail.com; 3 Clinical Development Services Agency-Centre of Clinical Research Excellence (CDSA-CCRE), JSS Hospital, JSS Academy of Higher Education and Research, Mahathma Gandhi Road, Mysore, Mysuru 570004, India; vinayagowd@gmail.com (V.R.G.); riyajohnson24@gmail.com (R.J.)

**Keywords:** digital health literacy, health literacy, older adults, ageing, rural

## Abstract

An ageing population, disproportionally affecting developing countries, increases demand on healthcare systems. Digital health offers access to healthcare for older people, particularly those residing in rural areas, as is the case for 71% of older adults in India. This research examined technology uptake and digital and health literacy (eHEALS) among a sample of 150 older adults in rural Mysore and Suttur, India. The study utilised mixed-method, with descriptive analysis of quantitative data and thematic analysis of qualitative data. Low rates of digital (11%) and health literacy (3–27% across domains) were identified. Mobile phone ownership was 50%, but very few owned or used a smartphone and less than 10% used the Internet to contact health professionals. Qualitative analysis found low technology usage, driven by limited exposure and confidence in using digital devices. Barriers to usage included poor traditional literacy and physical aspects of ageing like poor vision. Social support from neighbours, family and local primary healthcare staff may enable adoption of digital health. Access to healthcare through digital means among Indian rural older adults needs to consider low rates of both digital and health literacy and leverage the value of support from family and primary healthcare providers.

## 1. Introduction

The World Health Organisation anticipates that by 2050 the number of people aged over 60 years will have doubled to over two billion, up from 900 million in 2015 [1]. The effect of this demographic change will be experienced disproportionately, with the greatest increases in aged populations occurring in developing countries. In India, the number of people aged over 60 years is expected to increase to 315 million people by 2050, more than three times the number registered in 2011 [1,2]. Of concern are the potential economic and social implications that an increasing ageing population may have on communities and healthcare systems [2,3,4].

Ageing is associated with increased risk of chronic conditions such as hearing loss, diabetes, cardiovascular disease, chronic obstructive pulmonary disease, cataracts, Alzheimer’s and other dementias, depression and osteoarthritis [1]. These conditions challenge the independence and quality of life of older people [5], exerting a significant burden on individuals, communities and healthcare systems [2]. The resultant increases in chronic conditions and corresponding decrease in independence that will accompany a larger aged population are associated with increased utilisation of healthcare services, greater costs to the healthcare system and more pronounced inequalities for those who have difficulty affording or accessing healthcare [4,6,7,8]. Affordability and transport are already the top two barriers to health service access reported by older people in low to middle income countries [1,9]. This is particularly salient among those residing in rural areas, as health care services are often clustered in urban centres, requiring extensive travel for those visiting from smaller towns and communities [1,9]. Currently, around 71% of Indian older people live in rural areas [10]. With a large proportion of older adults already experiencing difficulties accessing healthcare due to health or geographic factors, and with this number set to increase with the ageing population, it is imperative to identify methods to bridge inequities in healthcare access for older adults and to alleviate rising costs to the healthcare systems.

The use of Information and Communication Technologies (ICTs), such as computers, smart phones, the Internet and other communication devices, for digital health or eHealth initiatives may provide opportunities to address the current and impeding challenges for access to health care in order to reduce health inequities [11]. As health and social care take advantage of the digital revolution, ICTs have been acknowledged as essential in the transformation of health care systems and the delivery of patient-centred care that overcomes challenges associated with ageing and caters to the needs of older adults [1,3]. ICTs may provide unique opportunities for equitable healthcare access to manage and improve the health and quality of life of older adults through remote monitoring, telemedicine and other internet based supports [12,13,14,15,16]. While the success of these digital technologies in improving, their ability to be impactful for health is reliant upon end user adoption [17,18], for which a person’s eHealth literacy, and underpinning digital literacy and health literacy, are pivotal [19].

eHealth literacy is defined as a person’s ability to locate, understand and interpret health information from electronic sources, and to utilise new knowledge in health-related management or decision making processes [19,20]. It is a multifaceted construct underpinned by six underlying forms of literacy, with these being traditional literacy (reading and writing), information literacy, media literacy, scientific literacy, health literacy and computer (digital) literacy [20]. Health and digital literacy are especially pertinent among older adults [19].

Health literacy refers to the ability of an individual to acquire and interpret health information, and the application of acquired knowledge to making appropriate health-related decisions. Among older adults, low health literacy is associated with poorer health management, including the adoption of preventative health behaviours, adherence to medication regimes and knowing when to seek medical care [21].

Digital literacy is defined as “the awareness, attitude and ability of individuals to appropriately use digital tools and facilities to identify, access, manage, integrate, evaluate, analyse and synthesize digital resources, construct new knowledge, create media expressions, and communicate with others, in the context of specific life situations, in order to enable constructive social action” [22]. An age-related digital divide exists, potentially disadvantaging older adults regarding the use of ICT to enable access to healthcare [23,24]. Despite the rapid increase in the size of the ageing population, older adults experience the lowest rates of the uptake of digital technologies [15,24].

**Figure 1:**
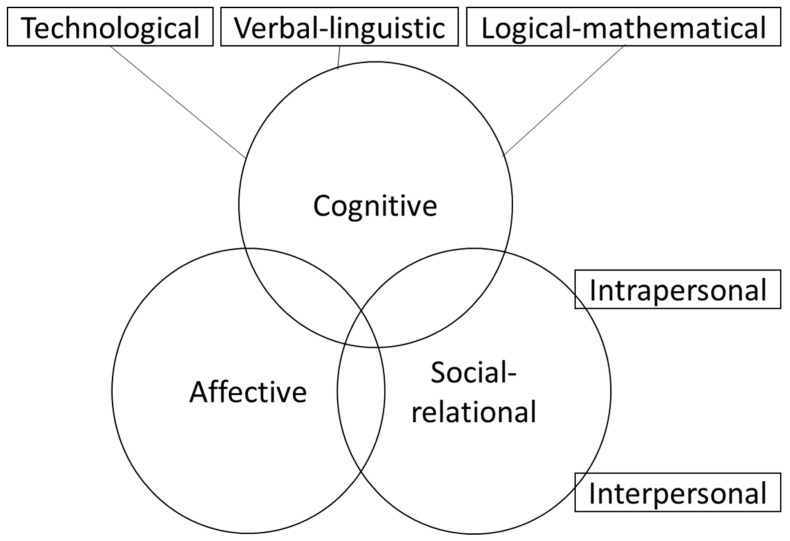
Digital literacy framework, adapted from Cartelli 2010.

Adoption of technology is influenced by a person’s skill and proficiency in using technology [25]. Cartelli 2010 proposed a framework for the adoption of digital technology (Figure 1), in which the adoption of technology is underpinned by three interacting main domains, namely (i) Cognitive domain, whose constituent elements include knowledge, comprehension, application, analysis, synthesis, evaluation; (ii) the Social-relational domain underpinned by introspective, and self-reflective skills and capacities; and (iii) the Affective domain—receiving phenomena, responding to phenomena, evaluating, organising and internalising phenomena [26]. In particular, the cognitive and affective domains may provide insight into the slow uptake of technology by older adults, due to limited technological exposure and application, inexperience and subsequent lack of confidence. As well as diminishing capacities for receiving and responding to phenomena, older people may be reluctant to engage in the use of technology [27,28].

Although largely skewed towards middle to upper income countries, the rise in the ageing population has a corresponding increase in the uptake of technology [3,29,30], and there is paucity of research pertaining to digital strategies for enabling the health of older adults [3]. Few studies have focused on the experiences of older people using technology [31], and there is limited understanding about how this group responds to and engages with technology. Additionally, only a small amount of research exists investigating the health literacy of older adult populations specifically, and current evidence is contradictive and fragmented [21]. Health initiatives that rely on digital solutions are likely to be underutilised if they do not meet the needs of the target audience, or if people do not have the skills or required literacies to engage in them. Thus, it is imperative to better understand levels of eHealth, digital and health literacies, as well as the needs from the end users’ perspectives, before technological strategies to improve access to healthcare can be implemented [32]. The aims of this study were to investigate the levels of eHealth, digital and health literacies among older people in rural India, and to understand how the population is positioned for a digital health world.

Ethical approval was granted by the JSS Medical College Institutional Ethics Committee (JSSM/IEC/2308/16/Clinical Study/2019-20) and registered with the La Trobe University Research Ethics Committee.

## 2. Material and Methods

### 2.1. Study Design and Setting

This was a mixed-method cross-sectional focus group and survey-based study that was performed in the rural city of Mysore and Suttur village, Karnataka, India. Mysore is home to JSS Hospital, an 1800-bed acute and outpatient ambulatory care teaching hospital. The hospital provides 37 specialties/super specialties and 67 specialist clinics. It caters to the healthcare needs of more than 16,000 outpatients and 18,000 inpatients every month. A significant number of patients come from the surrounding villages, including Suttur.

### 2.2. Participant Recruitment and Inclusion

Assuming 30% digital literacy [33] and a maximum margin of error of 8% at a 95% confidence interval, a minimum *n* = 127 participants were required. Participants included patients over 65 years who resided in rural settings in the JSS Hospital catchment and were invited to participate in the study. A team of three JSS research assistants and a volunteer who was a recently graduated medical practitioner recruited participants in the waiting rooms of JSS Hospital’s Medicine, Endocrinology, Geriatrics, Rheumatology, Gastroenterology, Pulmonology, Orthopaedics, Dermatology and Nephrology outpatient clinics from 5–19 September 2019. Prospective participants were provided with a participant information statement in English or Kannada language. The lead author provided detailed study briefing to the RAs interpreting between English and Kannada. Individuals willing to participate in the study provided signed consent.

### 2.3. Digital Literacy Model

The framework proposed by Cartelli, described earlier [26], was used as the foundation for data collection and analysis to guide in-depth assessment and understanding of the level of digital competency among the older people in our study. Although the development of the framework is traceable to an educational setting in a much younger population, the underlying principles that explain adoption and engagement with technology are not age or generation constrained. Thus, the model domains are relevant and translatable to older generations.

### 2.4. Quantitative Data Collection

Data were collected via a survey containing purpose-designed and standardised questionnaires, which was self-administered or administered with assistance from the RAs. The survey was available in English and Kannada. Prior to the study, an Australian professional translation agency was engaged to translate and back-translate the survey from English to Kannada. The JSS Hospital based study partners performed independent back-translation for quality assurance, adjudging the translation satisfactory.

The survey covered three categories: participant demographics, digital literacy and a health literacy screening tool (Supplementary Materials). Digital literacy assessment was based on the eHealth Literacy Scale (eHEALS) [19,20]. Response items focus on knowledge and frequency of usage of information and communications technologies for health purposes and general life tasks. The eHEALS had a high internal consistency in the current sample (Cronbach’s α = 0.93), which was comparable to reliability from previous studies [19,34]. Scores are summed to yield a total score of eight (low) to forty (high digital literacy). Health literacy was measured using the three health literacy screening questions [35] derived from the Short Test of Functional Health Literacy scale [36]. The questions have acceptable performance in detecting inadequate health literacy (AUC-ROC = 0.72 to 0.89) compared to the Rapid Estimate of Adult Literacy in Medicine tool as the standard [35]. Each item was analysed separately as recommended by the developers of the scale [35,36].

### 2.5. Qualitative Data Collection

After completing the survey, participants were invited to a focus groups (FG) moderated by author TR. Other senior members of the research team (IB and/or PP) were allowed interjections and clarification questions. Probing questions asked about participants’ self-management of their health, the role of family members in health management, health beliefs, sources of health information and support, use of digital technologies and support networks to facilitate health management and the use of digital technologies. Three focus groups were undertaken with eight, nine and eight participants, respectively. The fourth was a community forum (CF) with thirty-three participants. Discussions were audio-recorded and transcribed in English by VRG and RJ. TR and RM performed thematic analysis of the transcripts.

### 2.6. Data Analyses

Quantitative data analyses were undertaken in IBM SPSS statistics Version 25. Descriptive statistics comprised frequencies and proportions for categorical variables, and median and interquartile range (IQR) for non-normally distributed continuous variables. Associations between demographic characteristics and digital literacy and health literacy were analysed using non-parametric tests: Fisher’s exact test (gender and education with digital and health literacies), Mann–Whitney test (age with health literacy; gender and eHealth literacy; health literacy and eHealth literacy), Kruskal–Wallis test (associations between age and digital and health literacies, associations between education and eHealth literacy) and Spearman’s correlation (association between age and digital literacy).

Qualitative data were managed in NVivo and underwent inductive thematic analyses guided by the Cartelli digital literacy framework while open to emergent themes outside the framework domains. Raw data were broken down into the smallest meaningful segments of text and coded in alignment with the analysis framework. Prevalent and recurring themes were identified, compared and continuously refined in an iterative process. Analysis continued until the point of saturation was reached. Rigour of the findings was confirmed through independent analysis by two different researchers (TR and RM), followed by comparison and discussion of the respective findings.

## 3. Results

The demographic and health-related characteristics of the survey sample (*n* = 150) are presented in Table 1. Nearly two-thirds of participants were male (62.7% vs. 37.3% females), with the median age 71 years (65–99 years). Most participants (67.4%) had only primary education. The most reported current condition was diabetes mellitus (30%), followed by hypertension (18%) and eye or sight related conditions (14.7%). Whilst 79.3% of participants reported JSS Hospital to be the hospital they visited normally, 90% reported having a community health facility that was closer to their place of residence. Sixty-two percent of participants reported having two or more visits to JSS Hospital in the previous 12 months, with a median travel time of 45 (5–360) min.

### 3.1. Quantitative Analysis

The prevalence of technology usage and associations with sex and education are summarised in Table 2. A small proportion of participants reported having a home phone (6%), while more than half reported having a working mobile phone. Over one third of participants (37%) reported having problems with mobile phone reception. A significantly higher proportion of men reported owning mobile phones, using social media and using the internet to contact health professionals and access health information. Patients with education levels of secondary education of higher exhibited significantly higher mobile phone and other device ownership, computer usage, sending text messages, social media usage, etc.

The proportions of patients with adequate digital and health literacies, and associations between these and sex and education, are summarised in Table 3.

Only 10% of participants had used the Internet for shopping, banking and/or health related purposes. Less than four percent of participants had adequate health literacy relating to reading (i.e., nearly 97% of participants some of the time or more frequently). Approximately one quarter of patients had adequate health literacy relating to filling in forms (27.3%) and ability to understand written information (23.3%). However, this left approximately three quarters of participants with poor health literacy due to low confidence filling in forms (72.7%) and problems learning about medical conditions due to difficulty understanding written information (76.7%). Significantly higher proportions of men and people with higher levels of education displayed adequate digital and health literacies.

The median score for eHealth literacy was 24.0 (IQR = 0), and eHealth literacy was significantly associated with gender and education. Males exhibited significantly higher mean ranks for health literacy (79.72) compared to females (68.42, U= 2235.50, *p* = 0.016), whilst those with secondary education or higher had significantly higher mean ranks for health literacy (90.64) compared to those with primary education or less (68.15, U = 1732.50, *p* < 0.001). Those who had higher digital literacy, as defined by having used the Internet for online banking or shopping and/or for health-related purposes in the last month, also had higher eHealth literacy scores (124.44 vs. 69.66, U = 289.00, *p* < 0.001). Participants with adequate health literacy pertaining to learning about health conditions from written information (96.50 vs. 69.11, U = 1277.50, *p* < 0.001) and confidence in completing medical forms (96.30 vs. 67.67, U = 1381.50, *p* < 0.001) also had higher eHealth literacy compared to their respective counterparts.

### 3.2. Qualitative Analysis

Prominent themes from the focus groups are categorised into health behaviours and access to healthcare and digital literacy. These are summarised in Table 4 and described in detail below.

#### 3.2.1. Health Behaviours and Access to Healthcare

##### The Building of Health Beliefs and Pathways to Health

Participants identified the important role that food, remaining physically active and mental health played in overall health.

*“We eat healthy food like Ragi balls. We follow an active lifestyle. We go about looking after our livestock and toiling in the fields every day”… Patient 4, FG1.*

Family, friends and neighbours, as well as doctors, played an important role in building beliefs and reinforcing health behaviours and acted as conduits of health-related information. Television was also identified as a common medium through which healthcare information was obtained.

*“Our children tell us about eating habits and other lifestyle modifications. Sometimes we learn some things from the television”... Patient 1, FG3.*

*“When we fall sick, we go to doctor and they advise us to eat healthy food and do regular exercise”… Patient 22, CF4.*

*“We get to know about them from our friends and neighbours”… Patient 5, FG2.*

Participants were aware of distinctions between primary healthcare, which was provided at the local community/village level, and specialist care at a tertiary hospital in urban centres. Local primary health providers were the first point of contact for consultation relating to minor health-related issues, whereas more complex conditions were referred to specialists in larger urban hospitals.

*“There is one government run hospital for every 10 villages which has a doctor and a nurse. They refer us to the tertiary care centres in cities for any major health issues”… Patient 14, FG2.*

Participants valued the opportunity to consult with doctors face-to-face rather than receiving advice via telephone, particularly with regard to more complex medical conditions (described further under digital literacy). However, the nature of appointments in larger tertiary hospitals (whereby emergency cases were prioritised and waiting times could be long and unknown) was a source of concern, particularly for those who may have travelled significant amounts of time to attend an appointment.

*To get my FBS done I have to be on fasting 8 h and have to travel for four hours to reach hospital. The wait at the hospital OPD [out-patient department] increases the fasting duration to up to 10–12 h straight. In such cases, getting an appointment before coming to the hospital will be helpful”... Patient 3, FG2.*

#### 3.2.2. Digital Literacy

Findings relating to digital literacy are summarised within the context of the domains of the Cartelli digital literacy model: cognitive, social relational and affective.

##### Cognitive “Limited Exposure, Experiences and Efficacy with Technology”

Participants reported limited experience, knowledge and comprehension of technology, which was consistent with quantitative findings. Several participants identified having access to a home phone or, in some cases, access to a mobile phone including through a family member. However, their ability to utilise such technology to its full capacity was extremely limited.

*“Our children have mobile phones. I know how to talk using a phone, but I don’t know to make a call or use the keypad for messages”... Patient 4, FG3.*

As indicated, more participants had experience taking or making basic phone calls (i.e., talking to a health professional) compared to reading a text message. The underlying factors for this related to a lack of knowledge or comprehension of text messaging, or limited ability to read due to poor literacy or vision impairment, as described under the affective domain.

Only three participants indicated using the Internet, which they did in lieu of having to travel for second opinions for health-related matters. Interactions between the cognitive and social-relational domains underpinning digital literacy were evidenced through participants reporting that their children used the Internet or mobile phones to convey health information to their older parents.

*“We use internet to get health information instead of traveling for second opinion”… Patient 15, CF4.*

*“My son will browse the internet for information regarding health issues and explain it to me”… Patient 2, FG1.*

### 3.3. Social Relational Domain “It Takes a Village to Raise an Elder”

Multiple people within an older adult’s village are important contributors to the development of health beliefs, behaviours and maintenance of health. As highlighted earlier, interactions between neighbours, primary healthcare staff and, notably, family members were common means of accessing and interpreting health advice. The family and non-family social networks were crucial elements to navigating the health care system.

*“At home our families help us in taking care of our health. But they are busy today; hence we neighbours came together to the hospital”… Patient 6 & 5, FG3.*

*“Our friends and neighbours tell us about the health complications. They tell us about their experience regarding their surgeries, their admissions and what the doctor advised to prevent the complications”… Patient 25, CF4.*

Furthermore, interactions with family members facilitated familiarity with and access to technology for the older adults. This highlighted the role of family connections and interactions as a potential enabler to offset low digital and health literacy for improved use of digital health technologies among older adults.

*“My granddaughter is educated and sending messages will be helpful for me since I can’t read messages”… Patient 5, SG3.*

*“Sending a reminder message to my son’s phone regarding my appointment will be very helpful”… Patient 3, SG.*

Despite the above, older adults were mindful that they might be an undue burden to those they relied on for assistance relating to technology use or access to technology mediated healthcare services.

*“I cannot ask someone to read it out to me all the time. They might get irritated or annoyed after a couple of times. So we prefer calling [the hospital] sometimes”… Patient 7, SG4.*

### 3.4. Affective Domain—Lack of Confidence with Using Technology

The dominant factor in the affective domain was a lack of confidence in using technology. Many participants stated that they were not confident to receive health information via digital mediums due to their limited digital and health literacy making them unable to receive and interpret the information. Difficulties using technology further compounded concerns regarding the information required for more complex medical conditions.

*“If you can’t get it, you can’t use it”*

The lack of awareness of what was available with regard to digital health technology and the inability to simply receive health information via digital means were highlighted as barriers to usage. Several contributing factors were identified, including poor traditional literacy levels (some participants could not read), unavailability of devices that worked in the patient’s native tongue or the physical aspects of ageing, specifically deteriorating eyesight.

*“We don’t know how to read”... Patients 28 & 30, CF4.*

*“I know how to read and text but I can’t read because I can’t see. If my vision was good then I could read and text but now I can’t”… Patient 7, FG4.*

## 4. Discussion

This research investigated levels of digital and health literacies among older people in rural India to understand how these older people are positioned within a digital health world. Such information is vital to inform the development of user-friendly, simple digital initiatives that have the potential to bridge inequalities in access to healthcare services, particularly within the context of an increasing ageing population, a high proportion of whom reside in rural areas. Our findings revealed limited usage of communication technologies, and low health and digital literacies among the rural older adults in India. Strong social interactions with family, friends, neighbours and primary healthcare providers may serve as enablers for the adoption of simple digital initiatives.

More than half of our participants reported owning mobile phones. However, most of the devices were basic mobile phones with call and text capabilities only, rather than smartphones. Fewer participants reported being able to utilise text messaging, with most only confident in their abilities to make and receive phone calls. Less than ten percent of patients reported having an Internet connection at home and/or use of a computer. The limited use of technology in the current sample is consistent with previous findings [37], and may explain the low rates of technology usage for activities of daily living. Limited usage of digital technology is often underpinned by poor digital literacy [27,38]. Consistent with this, our participants displayed low levels of digital literacy, with only ten percent being identified as “digitally literate” based on having used a mobile or computer to access the Internet for online shopping, banking or healthcare related reasons. Low rates of technology usage and poor digital literacy have been described among older adults in varying contexts, contributing to a digital divide between older people and their younger counterparts [31]. We also identified lower health literacy among the current sample across all three domains. Both digital and health literacy were significantly associated with lower eHealth literacy scores and the latter two factors are important underpinnings of the broader concept of eHealth literacy [20]. Patients with higher eHealth literacy are better able to locate, scrutinise and apply health-related information for health self-management and have better patient-physician interactions, and initiatives aimed to improve eHealth literacy and those designing or applying technologies that suit the needs of older adults may provide substantial benefits to healthcare and health [38].

Our study participants described a holistic view of health, in which food, remaining active and looking after one’s mental health underpinned health and well-being, with medicines required if one should become sick. Whilst minor ailments were addressed locally by primary care physicians, more complex conditions were referred to specialists in larger urban centres. Accessing these centres was associated with lengthy travel and/or waiting times, posing an inconvenience to older adults and their supports. However, despite the inconvenience, participants still expressed a preference for seeing specialists face-to-face when dealing with more complex medical conditions. Very few participants identified the benefits of using the Internet to access health information in lieu of extensive travel, highlighting the paradox of digital divide versus the potential opportunity to exploit digital health technologies.

The lack of exposure to and confidence with technology, coupled with issues of ageing and deteriorating senses and low literacy, were some of the drivers of low rates of digital literacy and technology usage among this sample, potentially exacerbating reticence by Indian rural older adults to adopt digital health technologies. Nonetheless, this was mitigated by dependence on family and other social networks and, to some extent, local primary healthcare providers, underscoring the important role of these elements to any future success of digital heath models. That is, such models will need to consider simple technologies and recognise the social spheres of influence and collaborative supports of the older adults. In consideration of the desire to see a physician “face-to-face,” video conferencing to facilitate communication with urban specialists may provide a “best of both worlds” approach to ameliorating lengthy travel and wait times with the simplicity of a phone call but the added benefit of visualising the practitioner. These virtual consultations could be provided through local healthcare facilities and incorporate the primary healthcare provider to support both the use of the technology [39] as well as the patient’s comprehension of the information being provided. The adoption of telehealth in other populations has yielded successful results, providing cost-effective access to health care for geographically isolated people, as well as providing multidisciplinary, collaborative and streamlined care, patient empowerment and satisfaction with the use of tele-healthcare [40,41,42,43].

Of notable importance were the associations between sex, education and technology usage, digital literacy and health literacy. Similar to previous findings [37], men and those with higher levels of education exhibited higher usage of technology and digital and health literacy. Whilst our study did not allow for multivariable modelling, at the bivariate level, men displayed significantly higher levels of education compared to women, and it is likely that education is the mechanism behind the disproportionate adoption of technology and digital and health literacies between the sexes. Widespread improvements in education may, therefore, facilitate better health and digital literacies and contribute to the overall health of Indian older adults.

The findings of this study should be considered within the context of several limitations. The current sample did not allow for multivariable modelling; thus, the adjusted effects of sex and education on technology use and digital and health literacies were not able to be investigated. Additionally, our cross-sectional methodology is unable to attribute causation. Finally, qualitative data collection occurred in English, via the use of a translator. Thus, it is possible that the meaning and interpretation of questions posed to participants was not as intended. Our qualitative findings were, however, consistent with those from our quantitative data collection, as well as findings from previous existing literature. Finally, the large number of participants engaged provided a diverse range of representative views.

## 5. Conclusions

We found low rates of technology adoption, as well as poor digital, health and eHealth literacy, among the current sample with limited experience and exposure to technology and low confidence contributing to this phenomenon. Strong social support from family and local primary healthcare providers are potential enablers for future considerations to use technology to bridge inequities in healthcare, coupled with the adoption of a simple technology that overcomes poor literacy and physical aspects of ageing as barriers to technology use.

## Figures and Tables

**Table 1:** Demographic and health characteristics of survey respondents.

	*n*	%
Age (median (IQR), years	71 (68–78)	
Gender		
Male	94	62.7
Female	56	37.3
Highest level of education		
None	49	32.7
Primary	52	34.7
Secondary	29	19.3
Post-secondary	20	19.3
Participant self-reported presenting health conditions		
Diabetes mellitus	45	30.0
Hypertension	27	18.0
Fatigue	13	8.7
Eye/sight related condition	22	14.7
Myalgia/ joint pain/muscle pain	15	10.0
Pain, other parts of body	15	10.0
Loss of appetite	4	2.7
Hearing loss	6	4.0
Asthma	6	4.0
Other respiratory conditions	9	6.0
General age-related conditions	3	2.0
Cardiovascular related condition	5	3.3
Dermatological condition	8	5.3
Gastrointestinal condition	7	4.7
Fever	3	2.0
Other conditions	27	18.0
JSS Hospital as usual hospital visited		
Yes	119	79.3
No	29	19.3
Number visits to JSS Hospital in past 12 months		
0–1 visit	57	38.0
2–3 visits	27	18.0
4–5 visits	46	30.7
>5 visits	20	13.3
Travel time to JSS hospital (minutes) (median (IQR))	45.0 (70.0)	
Another hospital closer to residence		
Yes	135	90.0
No	15	10.0
Self-rated health [Median (IQR)]	5.0 (5.0)	

**Table 2:** Availability of or access to technology and technology use in last month for older adult patients from JSS hospital catchment area in rural India.

		Sex			Education		
	Total	Males	Females	*p*	≤Primary	≤Secondary	*p*
	*n* (%)	*n* (%)	*n*		*n*	*n*	
Availability/access to technology							
Home telephone							
No	141 (94.0)	87 (96.2)	54 (96.4)	0.49	96 (95.0)	45 (91.8)	0.48
Yes	9 (6.0)	7 (7.4)	2 (3.6)		5 (5.0)	4 (8.2)	
Mobile Phone							
No mobile phone	63 (42.0)	32 (34.4)	31 (55.4)	0.01	55 (55.0)	8 (16.3)	<0.001
Mobile phone (standard calls and texts)	73 (48.7)	49 (52.7)	24 (42.9)		45 (45.0)	28 (57.1)	
Smartphone with internet access	13 (8.7)	12 (12.9)	1 (1.8)		0	13 (26.5)	
Tablet							
No	145 (96.7)	89 (94.7)	56 (100.0)	0.16	100 (99.0)	45 (91.9)	0.04
Yes	5 (3.3)	5 (5.3)	0		1 (1.0)	4 (8.2)	
Problems with mobile phone coverage							
No problems	94 (62.7)	60 (63.8)	34 (60.7)	0.72	53 (55.2)	41 (83.7)	<0.001
Sometimes have problems	21 (14.0)	14 (14.9)	7 (12.5)		15 (14.9)	6 (12.2)	
Consistently have problems	35	20 (21.3)	15 (26.8)		33 (32.7)	2 (4.1)	
Internet access at home							
No	136 (90.7)	82 (87.2)	54 (96.4)	0.13	100 (99.0)	36 (73.5)	<0.001
Yes	14 (9.3)	12 (12.8)	2 (3.6)		1 (1.0)	13 (26.5)	
Used a computer							
No	146 (93.3)	91 (96.8)	55 (98.2)	1.00	101 (100.0)	45 (91.8)	0.004
Yes (home computer)	4 (2.7)	3 (3.2)	1 (1.8)		0	4 (8.2)	
Sent e-mail or text message							
Never	133 (88.7)	79 (84.0)	54 (96.4)	0.11	99 (98.0)	34 (69.4)	<0.001
Rarely	6 (4.0)	5 (5.3)	1 (1.8)		2 (2.0)	4 (8.2)	
Some days	7 (4.7)	7 (7.4)	0		0	7 (14.3)	
Most days	4 (2.7)	3 (3.2)	1 (1.8)		0	4 (8.2)	
Used social media							
No	137 (91.3)	82 (87.2)	55 (98.2)	0.03	101 (100.0)	36 (73.5)	<0.001
Yes		12 (12.8)	1 (1.8)		0	13 (26.5)	
WhatsApp	13 (8.7)						
Facebook	8 (5.3)						
Instagram	3 (2.0)						
Used internet for videoconferencing or communication							
No	148 (98.7)	2 (2.1)	0	0.53	0	2 (4.1)	0.11
Yes	2 (1.3)	92 (97.9)	56 (100.0)		101 (100.0)	47 (95.9)	
Used internet to shop for groceries/ personal items							
No	146 (97.3)	4 (4.3)	0	0.30	0	4 (8.2)	0.01
Yes	4 (2.7)	90 (95.7)	56 (100.0)		101 (100.0)	45 (91.8)	
Pay bills/banking							
No	146 (97.3)	90 (95.7)	56 (100.0)	0.30	101 (100.0)	45 (91.8)	0.01
Yes	4 (2.7)	4 (4.3)	0		0	4 (8.2)	
Contact/find health care provider							
No	134 (89.3)	79 (84.0)	55 (98.2)	0.006	101 (100.0)	33 (67.3)	<0.001
Yes	16 (10.7)	15 (16.0)	1 (1.8)		0	16 (32.7)	
Get information about health conditions							
No	144 (96.0)	88 (93.6)	56 (100.0)	0.08	101 (100.0)	43 (87.8)	0.001
Yes	6 (4.0)	6 (6.4)	0		0	6 (12.2)	
Order/refill prescriptions							
No	147 (98.0)	91 (97.8)	56 (100.0)	0.52	101 (100.0)	2 (4.2)	0.10
Yes	2 (1.3)	2 (2.2)	0		0	46 (95.8)	

**Table 3:** Participants’ digital and health literacy levels.

			Sex					Education Level				
	*n*	%	Male		Female			≤Primary		≤Secondary		
			*n*	%	*n*	%	*p*	*n*	%	*n*	%	*p*
Digital literacy							0.006					<0.001
Has NOT used internet for online shopping/banking or health-related purposes.	134	89.3	79	84.0	55	98.2		101	100.0	33	67.3	
Has used internet for online shopping/banking AND/OR health-related purposes.	16	10.7	15	16.0	1	1.8		0	0.0	16	32.7	
Health literacy												
Reading							0.62					0.04
Adequate	5	3.3	3	3.2	2	3.6		1	1.0	4	8.2	
Inadequate	145	96.7	91	96.8	54	96.4		100	99.0	45	91.8	
Learning							0.001					<0.001
Adequate	35	23.3	30	31.9	5	8.9		5	5.9	29	59.2	
Inadequate	115	76.7	64	68.1	51	91.1		95	94.1	20	40.8	
Forms							0.002					<0.001
Adequate	41	27.3	34	36.2	7	12.5		11	10.9	30	61.2	
Inadequate	109	72.7	60	63.8	49	87.5		90	89.1	19	38.8	

**Table 4:** A summary of major qualitative themes mapped to the Digital Literacy Framework domains and other.

Framework Domain	Theme
Cognitive	Limited exposure, experiences and efficacy with technology—thereby limiting usage of available technologies
Social relations	“It takes a village to raise an elder”—multiple people within an older adult’s village are important contributors to the development of health beliefs, behaviours and maintenance of health
Affective	Lack of confidence with using technology—resulting in limited capability to receive and interpret the information
Other	Health behaviours and access to healthcare—the building of health beliefs and pathways to health comprised of a multifactorial layer of family, friends, neighbours and doctors

## Data Availability

The data presented in this study are available on request from the corresponding author. The data are not publicly available to protect the privacy and confidentiality of the research participants.

## Supplementary Materials

The following supporting information can be downloaded at: https://www.mdpi.com/article/10.3390/geriatrics7020028/s1.
