# Supplementary material for: Exploring Telehealth Readiness in a Resource Limited Setting: Digital and Health Literacy among Older People in Rural India (DAHLIA)
Source: Geriatrics (Basel). 2022 Mar 1;7(2):28. doi: 10.3390/geriatrics7020028 (PMC8938771; doi:10.3390/geriatrics7020028)
Supplement: Supplementary file 1 [file geriatrics-07-00028-s001.zip › geriatrics-1599669-supplementary.pdf]

Supplementary Material: DAHLIA Project Survey

1. Demographic and characteristics data collection form

2. Age: __________ Gender: _____________

3. Education level:

   ☐None

   ☐Primary

   ☐Secondary

   ☐Post-secondary

4. What medical/health condition do you currently have?

5. Is this the hospital you normally visit for your health condition? ☐Yes ☐No

6. In the past 12 months, how many times have you visited this hospital?
   ☐ 0-1 Visit
   ☐ 2-3 Visits
   ☐ 4-5 Visits
   ☐ More than 5 visits

7. How many minutes do you travel to get to this hospital?

8. Is there another hospital or health facility closer to where you live? ☐Yes ☐No

9. Self-rating of current health status (please put a mark on the line to best represent how you currently feel about your health):

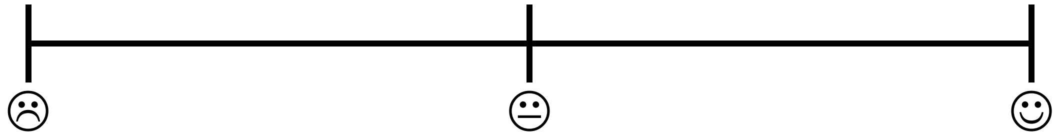

Very Poor | Neither Poor nor Good | Very Good

2. Digital literacy survey – knowledge and usage of information and communications technology

This survey is about knowledge and use of technology. Please use a tick to indicate your response

1. Do you have a working home telephone? ☐YES ☐NO

2. Do you have a working mobile phone?

   ☐NO
   ☐YES - I have a standard mobile phone for calls and texts
   ☐YES - I have a smartphone with Internet access

3. Do you have a working electronic tablet device (e.g. iPad)?
   ☐NO
   ☐YES, iPad
   ☐YES, Android
   ☐YES, Windows or another platform

4. If you have a mobile phone or tablet with cellular connectivity, do you have problems with lack of mobile phone service in your home? (e.g. a "blackspot" in which you cannot connect online by mobile phone or tablet)
   ☐NO, I can easily connect via mobile
   ☐YES, I sometimes have problems with mobile coverage
   ☐YES, I consistently have problems with mobile coverage

5. Have you used a computer in the past month?
   ☐NO
   ☐YES, I used my home computer
   ☐YES, I used a computer outside my home (e.g. at library or community centre)

6. Do you have Internet access at home?
   ☐NO
   ☐Yes, wi-fi
   ☐Yes, broadband
   ☐Yes, other type of access

7. Have you sent messages by e-mail or text message within the past month?
   ☐Never
   ☐Rarely
   ☐Some days
   ☐Most days

8. Have you used social media in the past month? (Check all social media types used)
   ☐I have not used social media
   ☐Facebook
   ☐Twitter
   ☐Skype
   ☐Instagram
   ☐Linked In
   ☐WhatsApp
   ☐Other (please specify):

9. In the past month, have you used the internet to:
Communicate with others (e.g. by Skype, Viber, FaceTime or other social media) ☐YES ☐NO

Shop for groceries or personal items	☐YES	☐NO
Pay bills or do banking	☐YES	☐NO
Contact or find any health care provider	☐YES	☐NO
Get information about health conditions	☐YES	☐NO
Order or refill prescriptions	☐YES	☐NO

10. For each statement, tell me which response best reflects your opinion and experience right now.

	Strongly Disagree	Disagree	Undecided	Agree	Strongly Agree
i. I know **what** health resources are available on the Internet	☐	☐	☐	☐	☐
ii. I know **where** to find helpful health resources on the Internet	☐	☐	☐	☐	☐
iii. I know **how** to find helpful health resources on the Internet	☐	☐	☐	☐	☐
iv. I know **how to use** the Internet to answer my questions about health	☐	☐	☐	☐	☐
v. I know how to use **the health information** I find on the Internet to help me	☐	☐	☐	☐	☐
vi. I have the skills I need to **evaluate** the health resources I find on the Internet	☐	☐	☐	☐	☐
vii. I can tell **high quality** health resources from **low quality** health resources on the Internet	☐	☐	☐	☐	☐
viii. I feel **confident** in using information from the Internet to make health decisions	☐	☐	☐	☐	☐

Appendix 3. Health Literacy Screening Questions

1. How often do you have someone (like a family member, friend, hospital/clinic worker or caregiver) help you read hospital materials? (**Help Read**)

   - ☐ All of the time
   - ☐ Most of the time
   - ☐ Some of the time
   - ☐ A little of the time
   - ☐ None of the time

2. How often do you have problems learning about your medical condition because of difficulty understanding written information? (**Problems Reading**)

   - ☐ All of the time
   - ☐ Most of the time
   - ☐ Some of the time
   - ☐ A little of the time
   - ☐ None of the time

3. How confident are you filling out forms by yourself? (**Confident with Forms**)

   - ☐ Always
   - ☐ Often
   - ☐ Sometimes
   - ☐ Occasionally
   - ☐ Never
